# A panel of genotypically and phenotypically diverse clinical *Acinetobacter baumannii* strains for novel antibiotic development

**DOI:** 10.1128/spectrum.00086-24

**Published:** 2024-06-25

**Authors:** Chunli Sun, Danyan Zhou, Jintao He, Haiyang Liu, Ying Fu, Zhihui Zhou, Sebastian Leptihn, Yunsong Yu, Xiaoting Hua, Qingye Xu

**Affiliations:** 1 Department of Infectious Diseases, Sir Run Run Shaw Hospital, Zhejiang University School of Medicine, Hangzhou, China; 2 Key Laboratory of Microbial Technology and Bioinformatics of Zhejiang Province, Hangzhou, China; 3 Regional Medical Center for National Institute of Respiratory Diseases, Sir Run Run Shaw Hospital, School of Medicine, Zhejiang University, Hangzhou, China; 4 Zhejiang University-University of Edinburgh (ZJU-UoE) Institute, Zhejiang University, Haining, Zhejiang, China; 5 Zhejiang Provincial People’s Hospital, People’s Hospital of Hangzhou Medical College, Hangzhou, Zhejiang, China; 6 Department of Clinical Laboratory, Sir Run Run Shaw Hospital, Zhejiang University, Hangzhou, China; 7 Key Laboratory of Precision Medicine in Diagnosis and Monitoring Research of Zhejiang Province, Hangzhou, China; 8 Department of Antimicrobial Biotechnology, Fraunhofer Institute for Cell Therapy & Immunology (IZI), Leipzig, Germany; 9 Department of Biochemistry, Health and Medical University, Erfurt, Germany; Universidad Nacional Autonoma de Mexico-Campus Morelos, Cuernavaca, Mexico

**Keywords:** *Acinetobacter baumannii*, heterogeneous, panel, novel antibiotic development, genomic analysis, phenotypes

## Abstract

*Acinetobacter baumannii* is one of the most important pathogens worldwide. The intrinsic and acquired resistance of *A. baumannii*, coupled with the slow pace of novel antimicrobial drug development, poses an unprecedented and enormous challenge to clinical anti-infective therapy of *A. baumannii*. Recent studies in the field of pathogenicity, antibiotic resistance, and biofilms of *A. baumannii* have focused on the model strains, including ATCC 17978, ATCC 19606, and AB5075. However, these model strains represent only a limited portion of the heterogeneity in *A. baumannii*. Furthermore, variants of these model strains have emerged that show significant diversity not only at the genotypic level but also reflected in differences at the phenotypic levels of capsule, virulence, pathogenicity, and antibiotic resistance. Research on *A. baumannii*, a key pathogen, would benefit from a standardized approach, which characterizes heterogeneous strains in order to facilitate rapid diagnosis, discovery of new therapeutic targets, and efficacy assessment. Our study provides and describes a standardized, genomically and phenotypically heterogeneous panel of 45 different *A. baumannii* strains for the research community. In addition, we performed comparative analyses of several phenotypes of this panel. We found that the sequence type 2 (ST2) group showed significantly higher rates of resistance, lower fitness cost for adaptation, and yet less biofilm formation. The Macrocolony type E (MTE, flat center and wavy edge phenotype reported in the literature) group showed a less clear correlation of resistance rates and growth rate, but was observed to produce more biofilms. Our study sheds light on the complex interplay of resistance fitness and biofilm formation within distinct strains, offering insights crucial for combating *A. baumannii* infection.

**IMPORTANCE:**

*Acinetobacter baumannii* is globally notorious, and in an effort to combat the spread of such pathogens, several emerging candidate therapies have already surfaced. However, the strains used to test these therapies vary across studies (the sources and numbers of test strains are varied and often very large, with little heterogeneity). The variation complicates the studies. Furthermore, the limited standardized resources of *A. baumannii* strains have greatly restricted the research on the physiology, pathogenicity, and antibiotic resistance. Therefore, it is crucial for the research community to acquire a standardized and heterogeneous panel of *A. baumannii*. Our study meticulously selected 45 diverse *A. baumannii* strains from a total of 2,197 clinical isolates collected from 64 different hospitals across 27 provinces in China, providing a scientific reference for the research community. This assistance will significantly facilitate scientific exchange in academic research.

## INTRODUCTION

*Acinetobacter baumannii* is highly viable pathogen that is widely distributed not only in the environment but also in the hospital, where it is an important causative agent of hospital-acquired infections. *A. baumannii* belongs to the notorious ESKAPE (*Enterococcus faecium*, *Staphylococcus aureus*, *Klebsiella pneumoniae*, *A. baumannii*, *Pseudomonas aeruginosa*, and *Enterobacter* spp.) group of microorganisms. High morbidity and mortality rates due to this group of bacteria place a great burden on healthcare systems worldwide (1). It is estimated that 45,900 people in the United States are infected with *A. baumannii* each year (2), and the mortality rate from *A. baumannii* infections in intensive care units can be as high as 54% (3).

Carbapenems are often used as the first-line drugs in the treatment of multidrug resistance (MDR) to *A. baumannii*; however, in recent years, carbapenem-resistant *A. baumannii* (CRAB) have been frequently reported, with the overall resistance rates showing a worrying increase (4). In 2018, the World Health Organization (WHO) evaluated 25 resistance patterns in 20 bacterial species based on 10 criteria such as mortality, healthcare burden, community burden, resistance prevalence, and 10-year resistance trends. The WHO ranked CRAB to be of critical priority, highlighting the urgent need for increased research and approaches that allow us to understand the pathogen and tackle infections in the clinic (5). The dissemination of CRAB is predominantly driven by a few dominant clones, including the ST2, ST1, ST79, and ST25 lineages, with ST2 predominantly present on a global scale (6). The main mechanism of carbapenem resistance in *A. baumannii* is the production of class D b-lactamases. *bla*_OXA-23_, *bla*_OXA-58_, and *bla*_OXA-24_ are the most common acquired carbapenemases in *A. baumannii*, and they vary between countries and regions, but are mainly dominated by *bla*_OXA-23_, which is located on chromosomes as well as on plasmids (7, 8). *bla*_OXA-23_ is mediated by Tn*2006*, Tn*2007*, Tn*2008*, Tn*2008B*, and Tn*2009* for translocation in CRAB, and there are geographical differences in the distribution of these transposons (9, 10).

The genome of *A. baumannii* is extremely plastic, with a conserved core genome estimated to account for only 14.76% (2,009/13,611) of the pan-genome, which is decreasing with the number of strains sequenced, indicating the extensive heterogeneity of *A. baumannii* strains (11). *A. baumannii* can acquire or maintain antibiotic resistance through insertion sequences, integrons, transposons, prophages, and other mobile elements (12). In addition, the natural transformation ability of *A. baumannii* contributes to its rapid acquisition of antibiotic resistance genes, virulence factors, and adhesion-associated factors, allowing the host to acquire new functions, that is, phenotypic heterogeneity (13, 14).

*A. baumannii* has a diverse population, with at least 237 capsular locus types and 22 lipooligosaccharide outer core types (15, 16). Current studies in the field of pathogenicity, antibiotic resistance, and biofilm of *A. baumannii* have focused on model strains such as ATCC 17978, ATCC 19606, and AB5075. However, these model strains represent only offer a glimpse into the heterogeneity of *A. baumannii*. Furthermore, variants of these model strains have emerged that show significant diversity not only at the genotypic level but also reflected in differences at the phenotypic levels of capsule, virulence, pathogenicity, and antibiotic resistance (17–19). More surprisingly, an IS*Aba13* insertion resulted in phenotypic changes that were distinct from the parental strain, and such insertional sequence transfer events are widespread (18). Therefore, continued use of these three limited strains is clearly inappropriate. We propose to make a collection of bacteria available to the community, which represents the wide heterogeneity of genotypes and phenotypes, to use this subset as a reference not only for scientific research but also for the rational treatment of *A. baumannii* infections.

In this study, we established a reference panel of 45 clinical isolates of *A. baumannii* based on ST Oxford typing analysis and described and evaluated their genotypic and phenotypic differences. Furthermore, we examined the strains within the panel for variations in genetic backgrounds and phenotypic traits, encompassing macrocolony morphology, resistance rate, biofilm formation, and growth rate. We hope that this panel collection reflects the wide diversity of *A. baumannii* strains and provides the scientific community with a scientific reference for development of novel drugs and therapeutic regimens against this species. At the same time, it can also serve as a database and collection to be used by researchers to quickly and rationally select model strains based on specific biological questions.

## MATERIALS AND METHODS

### Collection of a panel of *A. baumannii* strains

All *A. baumannii* strains were obtained from clinical specimens collected from 64 different hospitals in China between January 2009 and December 2010. A total of 2,197 non-duplicate *A. baumannii* strains were included, and they were widely distributed in 27 different provinces and regions, as described previously (20). Based on the diversity of the Oxford multilocus sequence typing (MLST) scheme, we ultimately selected 45 non-duplicate *A. baumannii* isolates representing the diversity of the collected species.

### Whole-genome sequencing and sequence analysis

Genomic DNA was extracted using a Qiagen Mini Kit (Qiagen, Hilden, Germany) and then sent for Illumina HiSeq (Illumina, San Diego, USA) sequencing and Oxford Nanopore MinION (Tianke, Zhejiang, China) sequencing. *De novo* genome assemblies of Illumina and Nanopore reads were performed using Unicycler v0.4.8 (21). The ResFinder database was used to identify antibiotic resistance genes using abricate v0.8.13 (https://github.com/tseemann/abricate), and the PubMLST database (https://github.com/tseemann/mlst) was used to identify sequence types, including Pasteur typing scheme and Oxford typing scheme. Bautype (22) was used to determine the types of capsule-encoding loci (KL) and lipooligosaccharide outer core loci (OCL). The pAci database was used to predict the replicons of each strain (23). Phylogenetic trees were constructed using RaxML v8.2.12 (24) and further visualized using iTOL v6 (25). Isolates were assessed for relatedness based on the core genomic SNP counts using snp-dists (https://github.com/tseemann/snp-dists). Breseq was employed to identify genes associated with tigecycline resistance and to detect mutations in the *gyrA* and *parC* genes that are linked to fluoroquinolone resistance phenotypes (26). The ISMapper tool was used in conjunction with IS*Aba1* sequences to assess the presence of IS*Aba1* located upstream of the *bla*_ADC_ gene (27). Recombinase recognition sites XerC/XerD (C/D) and XerD/XerC (D/C) were detected using pdifFinder (28). Sequence comparisons were performed and visualized using Easyfig 2.2.5 software (29). The map of China was visualized through the Hiplot Pro (https://hiplot.com.cn/) online website.

### Antimicrobial susceptibility testing (AST)

The minimal inhibitory concentrations (MICs) of cefepime, ceftazidime, imipenem, meropenem, ciprofloxacin, amikacin, gentamicin, colistin, eravacycline, and tigecycline were determined by the broth microdilution method according to the Clinical and Laboratory Standards Institute (CLSI) guidelines (30). *Escherichia coli* ATCC 25922 was used as the quality control strain in antimicrobial susceptibility testing. The breakpoints of tigecycline were interpreted following the guidelines of Food and Drug Administration (FDA) for *Enterobacterales*. The breakpoints for eravacycline were interpreted according to the guidelines of the European Committee on Antimicrobial Susceptibility Testing (EUCAST) for *Enterobacterales*, and the other antibiotics were interpreted according to the breakpoints recommended by (30), M100-S32) (30).

### Macrocolony morphology

Macrocolony morphology assays were performed as described previously (11) . Bacterial single colonies were selected and incubated overnight at 37°C at 200 rpm in Mueller–Hinton Broth (MHB). Five microliters of overnight bacterial suspension was added onto Columbia Agar containing 5% sheep blood (Becton, Dickinson and Company, Franklin Lakes, NJ). Plates were incubated non-inverted at room temperature for 6 days, followed by photographic recording with a camera. The experiment was biologically repeated three times to confirm the consistency of macrocolony morphology. Subsequently, a representative picture of each group of bacteria was selected and shown in Supplementary Material 1.

### Virulence in the *Galleria mellonella* infection model

The *G. mellonella* infection model was established as described previously (31). Briefly, overnight cultures of bacteria were washed with PBS and diluted to approximately 1 × 10^7^ CFU/mL. *G. mellonella* larvae were injected with 10 µL of the bacterial suspension into the first left prolegs (10 per group). Larvae were injected with 10 µL PBS as a combined trauma and solvent control. Incubation was carried out at 37°C in the dark. The experiment was repeated three times per group for 7 days. The number of surviving larvae was counted daily, and the log-rank test was performed using GraphPad Prism 8.4.3.

### Growth rate

Growth rates were determined as described previously (32). Three independent cultures of *A. baumannii* isolates were grown overnight and diluted to 1:100 in MHB, and then a 200-µL aliquot was placed into a flat-bottomed 100-well plate (triplicate experiments). The plates were incubated at 37°C. The optical density at 600 nm of each culture was measured every 5 minutes for 20 hours using a Bioscreen C MBR machine (Oy Growth Curves Ab Ltd., Finland). Growth rates were estimated from OD_600_ curves using an R script. ATCC17978 was used as a control for comparison with each isolate.

### Biofilm formation ability

Biofilm formation assays were performed as previously described, with minor modifications (33). Overnight cultures were diluted to 1:100 and transferred to 96-well cell culture dishes at 200 µL per well and incubated at 37°C for 24 hours. Each culture was added to three wells. The wells were washed three times with phosphate buffer solution (PBS) to remove unattached bacteria. The cells were stained with 0.1% crystal violet for 15 minutes and then washed with PBS to remove excess dye. Then, 95% ethanol was added and gently shaken for 20 minutes to release the dye, and the absorbance at OD_550_ was measured. Three independent experiments were carried out.

### Statistical analysis

Fisher’s exact test was used to compare the categorical variables between the two groups, and independent sample *t*-test was used to compare the differences between the sample means of the two groups. SPSS 21.0 software was used for data analysis, and *P* < 0.05 denotes statistically significant differences..

## RESULTS

### Diversity of the *A. baumannii* panel

**Fig 1:**
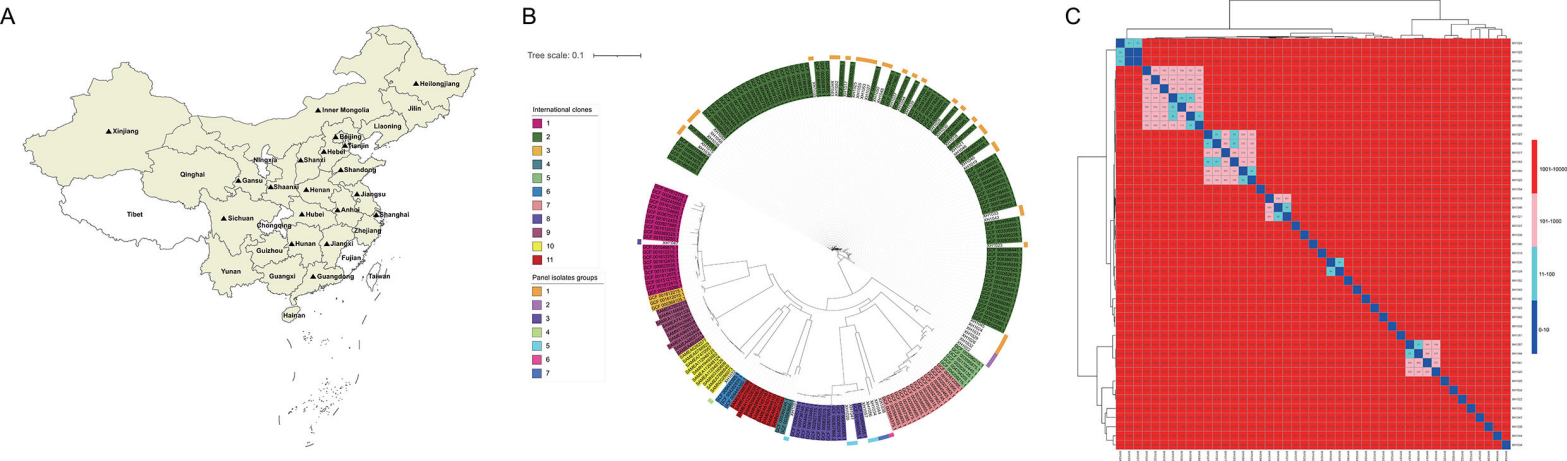
(**A**) Geographic distribution of clinical *A. baumannii* isolates collected in this study (beige area). The origin of the final strains included in the panel is indicated by filled black triangles. The source of this map is the Gaode Open Platform; (**B**) Phylogenetic tree of the panel strains with major human ICs. The ICs are shown in different colors, and the rings in the outer circle show the panel isolate groups; (**C**) Heatmap of 45 *A*. *baumannii* strains based on single-nucleotide polymorphisms (SNPs), and the distances of the SNPs between different strains are shown in the heatmap.

To maximize genetic diversity, 45 strains were selected as representatives of the large microbial diversity of *A. baumannii* based on ST Oxford typing. The strains comprising this panel were widely distributed, covering 19 provinces in China (Fig. 1A). By analyzing the genotypic and phenotypic differences of these 45 strains in terms of the antibiotic resistance profile, capsule type, and virulence phenotype, we obtained insights of the characteristics of the strains. We constructed a core phylogenetic tree of the strains in the final panel and those from the NCBI database representing the main international clones (ICs) prevalent in hospitals worldwide (IC1–IC11), with a total of 223 genomes included in this analysis. The phylogenetic tree showed that the strains in the panel formed seven groups: four clusters and three singletons. Cluster 1 and cluster 5 are closely related to IC2 and IC8, respectively, while singleton 3 clusters together with IC1 (Fig. 1B). In addition, there were eight strains that did not belong to any of the ICs depicted in this analysis. These strains are related to ICs, whereas they are too heterogeneous to be included in any major human ICs. Subsequently, we further analyzed the 45 *A*. *baumannii* strains based on single-nucleotide polymorphisms (SNPs) in the core genome, with a maximum difference of 58,373 SNPs. With the exception of individual strains, such as XH1024 with XH1029 and XH1031 from the same hospital in Guangdong province, the number of SNPs were less than 34, and the SNPs between XH1056 and XH1057 from Hebei province were 22, and the SNPs between XH1057 and XH1058 from Gansu province were 15, suggesting the existence of nosocomial transmission of these strains. The SNPs between the other strains were generally very far apart in the genome, indicating that the selection of the panel reaches a high level of heterogeneity, covering a wide diversity of strains (Fig. 1C).

**Fig 2:**
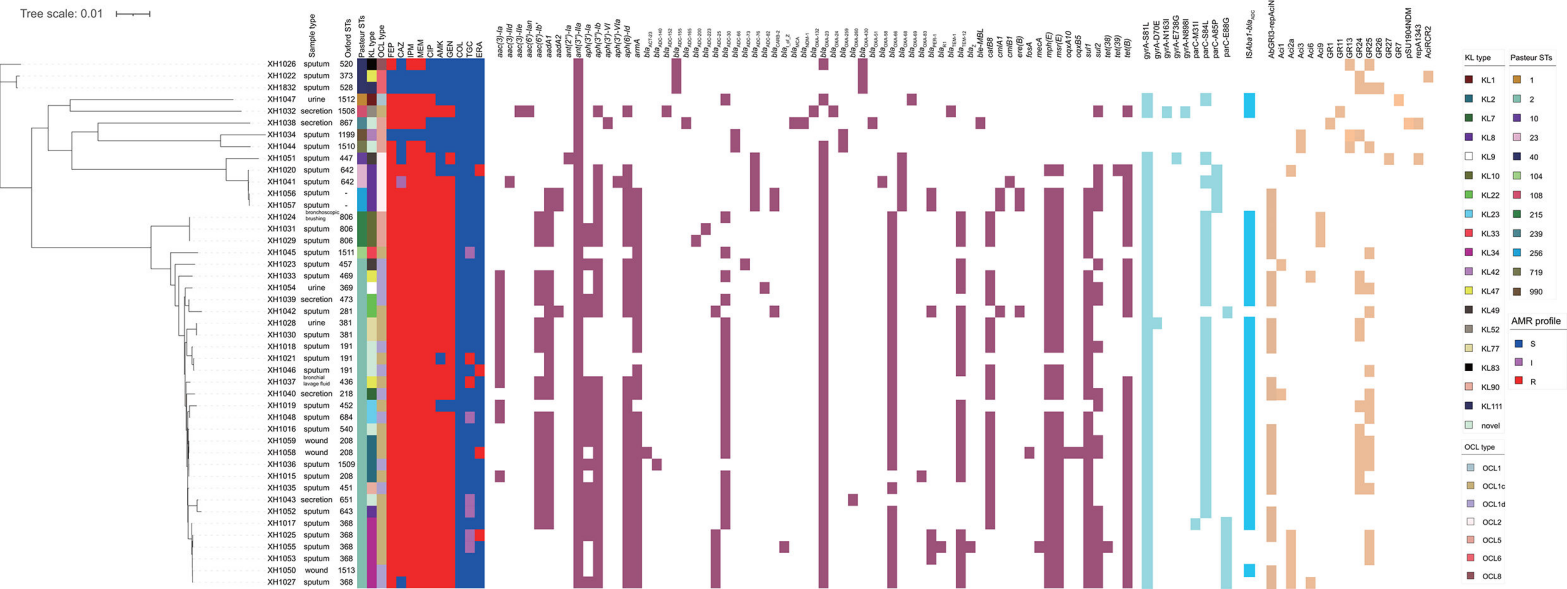
Characterization of the 45 *A*. *baumannii* strains. Phylogenetic tree constructed based on SNPs in the core genome of the panel strains. Sequence type (Pasteur scheme and Oxford scheme), KL type, OCL type, antibiotic sensitivity profile, ARGs, and plasmid types are listed. FEP, cefepime; CAZ, ceftazidime; IPM, imipenem; MEM, meropenem; CIP, ciprofloxacin; AMK, amikacin; GEN, gentamicin; COL, colistin; TGC, tigecycline; ERA, eravacycline.

The panel contains 31 known different Oxford STs as well as two strains (XH1056 and XH1057) that could not be classified as corresponding STs due to the lack of the Oxford scheme allele *gdhB*. According to the Pasteur scheme, the panel identified total 12 STs, including the most globally prevalent clone ST2, which is also overwhelmingly dominant in this panel (28/45), followed by ST40 (3/45) and ST215 (3/45). The panel contained 18 different capsule types known to be involved in capsule synthesis, with a further seven strains belonging to the novel KL type (7/45), which dominated the panel considerably, followed by KL34 (6/45), KL8 (5/45), and KL2 (4/45). Subsequent analysis of OCL loci revealed that OCL1c and OCL1d had the highest prevalence, accounting for 66.7% (30/45) of the whole panel isolates, followed by OCL5 (6/45) and OCL2 (2/45). Prediction of plasmid replicons (0–3 per strain) for this panel using the pAci database showed that AbGRI3-repAciN was most abundant, which was identified in 25 strains, while GR25 was found in 18 strains and GR24 in 15 strains (Fig. 2).

### AST results and antibiotic resistance genes (ARGs)

**Fig 3:**
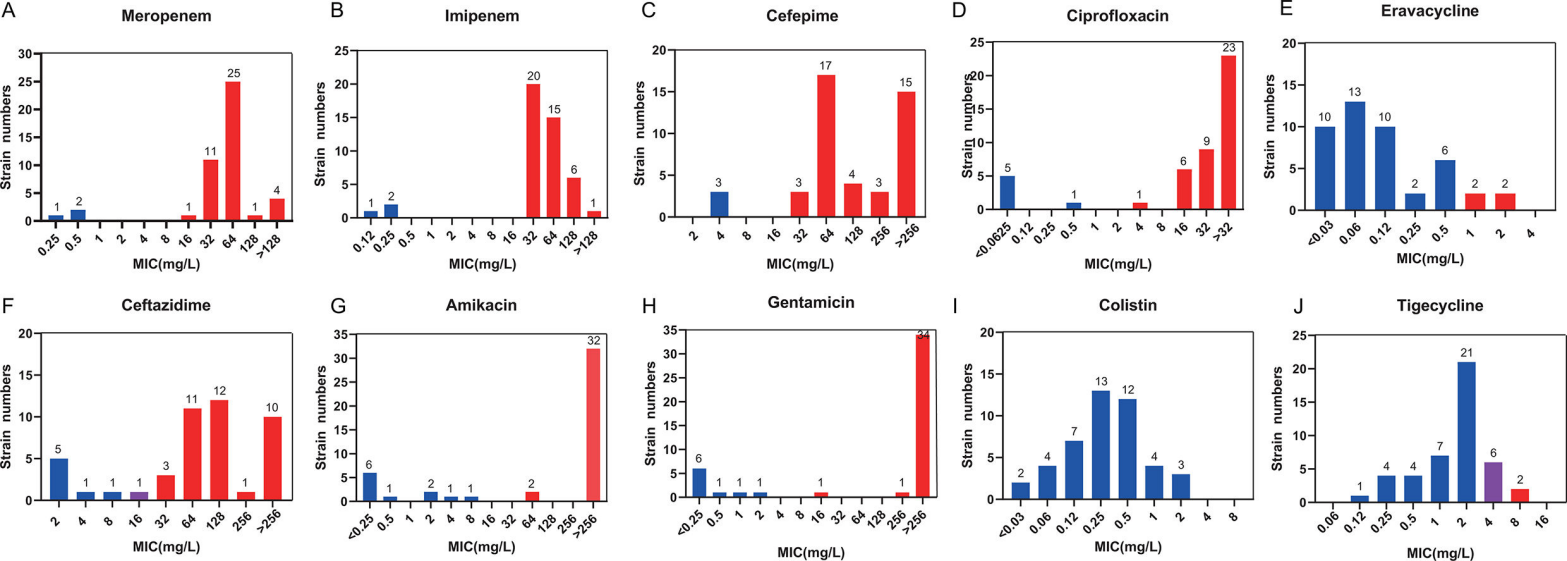
MIC distribution of panel strains against tested antibiotics. (**A–J**): MIC distribution of strains against meropenem, imipenem, cefepime, ciprofloxacin, eravacycline, ceftazidime, amikacin, gentamicin, colistin, and tigecycline respectively. Blue, purple, and red colors indicate the antimicrobial susceptibility, intermediary, and resistance phenotypes of each tested antibiotic, respectively.

The AST results showed that three isolates (XH1022, XH1832, and XH1034) were susceptible to all 10 antibiotics tested, and one isolate (XH1025) was non-susceptible to all tested antibiotics, except colistin. Specifically, 42 strains were resistant to carbapenems (imipenem and meropenem), and the minimum inhibitory concentrations were mainly in the range of 32–64 mg/L. Additionally, 36 strains were found to be resistant to aminoglycosides (amikacin and gentamicin), with 32 strains and 34 strains having extremely high MICs (MIC ≥256 mg/L) for amikacin and gentamicin, respectively; whereas 42 strains were identified as resistant to cephalosporins (ceftazidime and cefepime), and 39 strains were resistant to fluoroquinolones (ciprofloxacin). In contrast, resistance to tigecycline was very low (2/45, 4.4%), and only four strains were resistant to the new antimicrobial drug eravacycline. All strains in this panel were colistin-susceptible (Fig. 3A through J).

Overall, a variety of known ARGs were detected in this group of isolates, and a total of 65 different ARGs were found to be resistant to the six classes of antimicrobials (aminoglycosides, carbapenems, fluoroquinolones, tetracyclines, lipopeptides, and cephalosporins). All strains carried *ant (3'')-IIa*, *bla*_ADC_ and *bla*_OXA-51-like_ genes as expected, which are inherent to *A. baumannii*. The *bla*_OXA-51-like_ family detected *bla*_OXA-51_, *bla*_OXA-66_, *bla*_OXA-68_, *bla*_OXA-69_, *bla*_OXA-83_, *bla*_OXA-132_, *bla*_OXA-259_, *bla*_OXA-260_, and *bla*_OXA-430_, which also illustrates the significant variability among the strains in our collection. Of the 42 strains, carbapenemase genes were encoded by *bla*_OXA-23_ (*n* = 40), *bla*_OXA-24_ (*n* = 1), *bla*_OXA-58_ (*n* = 1), and *bla*_NDM-1_ (*n* = 1), with *bla*_OXA-23_ being the most common. One strain, XH1041, possessed two carbapenemase genes, *bla*_OXA-23_ and *bla*_OXA-58_. In addition to carbapenem resistance genes, strains also carried aminoglycoside resistance genes such as *aadA1*, *aph (3')-VIa*, *aph (3')-Ia,* and *aac (6')-Ib'*. In the diversity panel of this study, a total of 39 strains were found to be resistant to ciprofloxacin. All of these resistant strains exhibited amino acid alterations in either the DNA gyrase (GyrA) or topoisomerase IV (ParC). The most common mutation observed was a combination of mutations in both genes, specifically GyrA (S81L) and ParC (S84L), which has been confirmed by previous research as the primary cause of fluoroquinolone resistance in *A. baumannii* (34, 35). Furthermore, among these cephalosporin-resistant strains, 29 strains were found to have IS*Aba1* directly inserted upstream of the *bla*_ADC_ gene, a mechanism that has been confirmed in earlier research as conferring resistance (36). *A. baumannii* XH1021 and XH1047 are resistant to tetracycline-class third-generation derivative tigecycline. Genomic analysis detected the insertion of IS*Aba1* within the *adeN* gene of XH1037, which has been proven to confer tigecycline resistance (26). The mechanism of tigecycline resistance in XH1021, however, remains to be fully elucidated. In 31 strains, we detected the tetracycline resistance gene *tet(B*), while 37 strains contained the sulfonamide resistance genes *sul1* and *sul2* (details in Fig. 2).

### Carbapenem resistance gene carriage and plasmid characterization of different Oxford ST isolates

**Fig 4:**
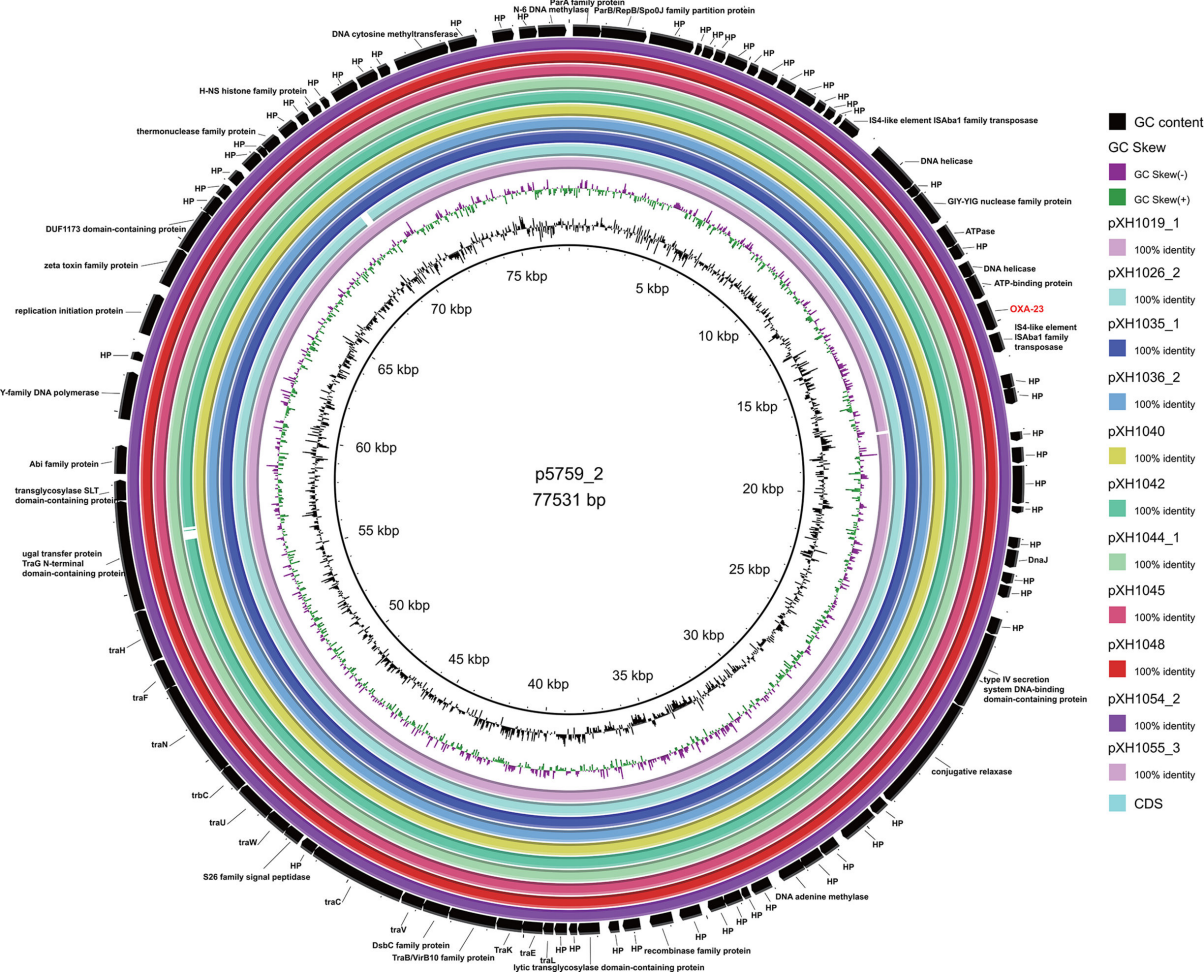
Using p5759_2 as a reference, BRIG comparisons were performed on pXH1019_1, pXH1026_2, pXH1035_1, pXH1036_2, pXH1040, pXH1042, pXH1044_1, pXH1045, pXH1048, pXH1054_2, and pXH1055_3, which showed high sequence similarity to p5759_2 isolated from Beijing.

**Fig 5:**
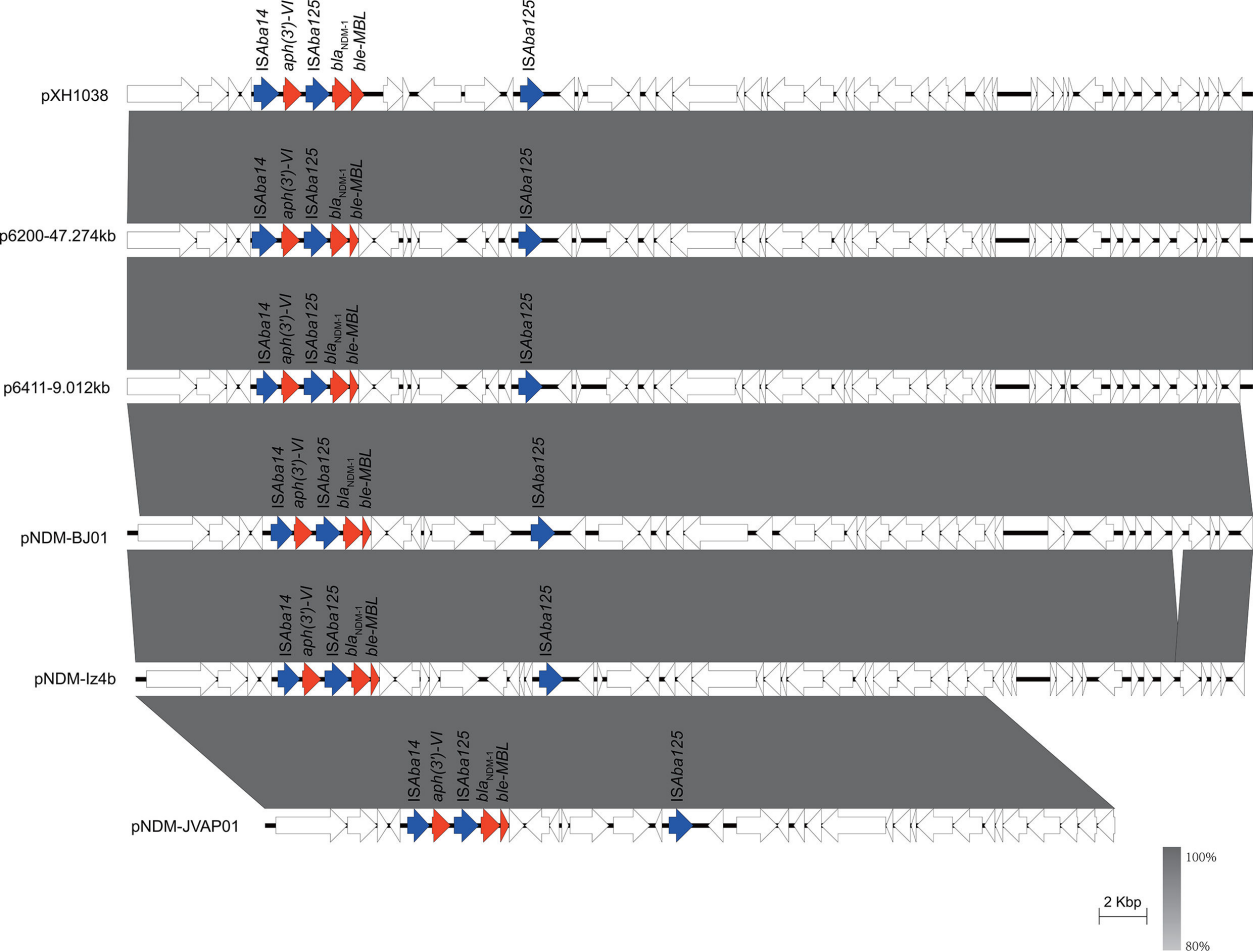
Scaled, linear sequence comparison of plasmids pXH1038, p6200-47.274kb (CP010399.1), p6411-9.012kb (CP010370.2), pDDM-BJ01 (JQ001791.1), pNDM-lz4b (KJ547696.1), and pNDM-JVAP01 (KM923969.1). Resistance genes are shown as red arrows, insertion sequence-related genes are shown as blue arrows, and other genes are shown as white arrows.

**Fig 6:**
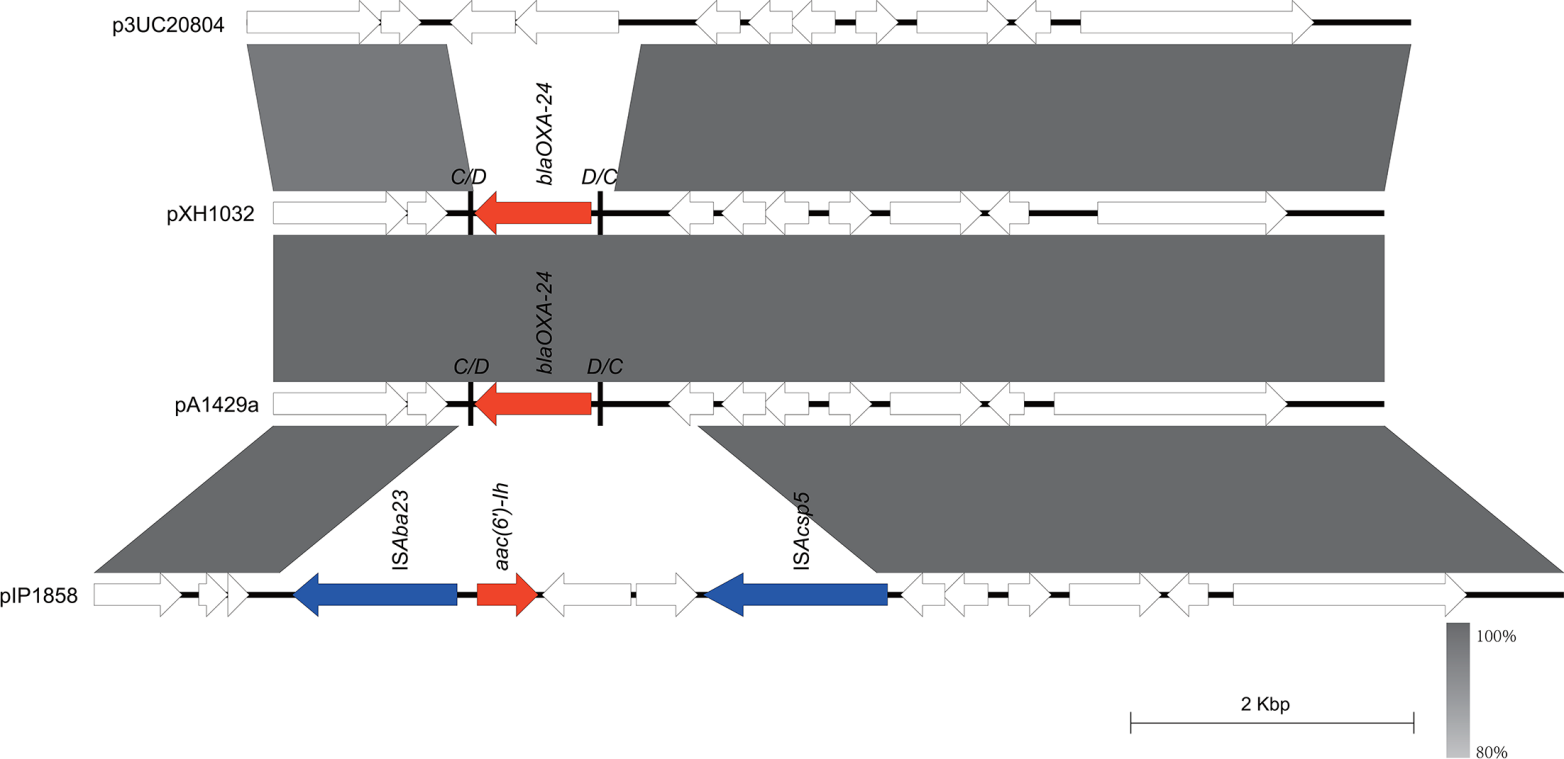
Scaled, linear sequence comparison of plasmids pXH1032, p3UC20804 (CP076810.1), pA1429a (CP046901.1), and pIP1858 (KP890934.1). Resistance genes are shown as red arrows, insertion sequence-related genes are shown as blue arrows, and other genes are shown as white arrows.

**Fig 7:**
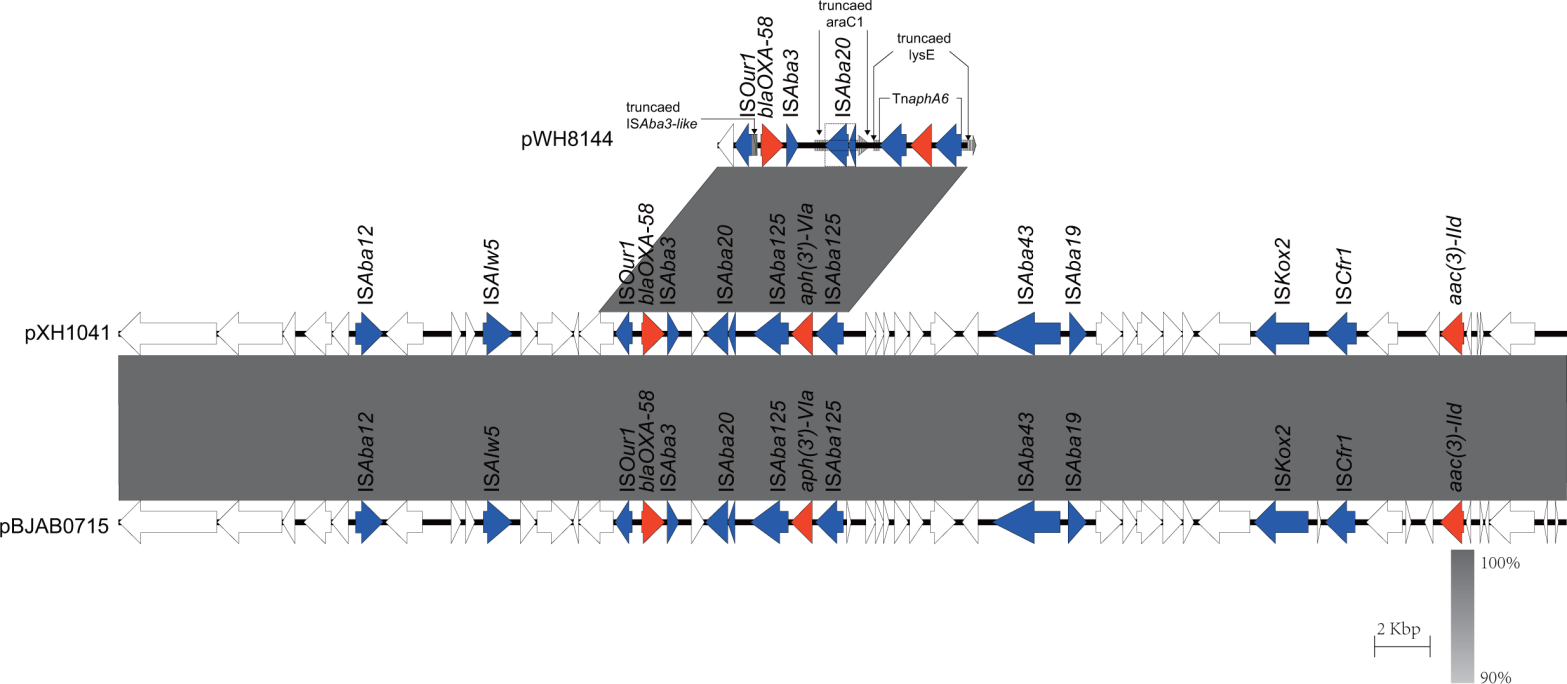
Scaled, linear sequence comparison of plasmids pXH1041, pBJAB0715 (CP003848.1), and the partial sequence of plasmid pWH8144 (Q241792.1). Resistance genes are shown as red arrows, insertion sequence-related genes are shown as blue arrows, and other genes are shown as white arrows.

Next, we selected a representative strain from each of the different Oxford STs and the undefined ST (XH1056), totaling 32 strains, with which we perform nanopore sequencing. Most of the *bla*_OXA-23_ genes were located in Tn*2009* (24/27) and half on chromosomes (16/27). The majority of isolates carried one copy of *bla*_OXA-23_, approximately 66.7%, followed by two copies (29.6%) and four copies (3.7%). The MICs of imipenem and meropenem ranged from 32 to >128 mg/L, respectively, and there was no correlation between the MICs of carbapenem antibiotics and the number of copies of *bla*_OXA-23_. Multiple copies of *bla*_OXA-23_ readily aggregated in tandem and also inserted independently into different genomic loci (Table 1). Isolate XH1037 carries a *bla*_OXA-23_ gene on Tn*2008*; isolates XH1023 and XH1024 carry *bla*_OXA-23_ on Tn*2006*, and all of them embedded in the chromosomes. Isolates XH1019, XH1026, XH1035, XH1036, XH1040, XH1042, XH1044, XH1045, XH1048, XH1054, and XH1055 all carry *bla*_OXA-23_ on a GR25 plasmid (Fig. 4), which shares high sequence similarity with p5759_2 (CP096712.1). The *bla*_NDM-1_ gene in pXH1038 is associated with a single copy of IS*Aba125*. Comparative analysis of plasmids reveals that pXH1038 shares a high degree of sequence similarity with plasmids p6200-47.274 kb (CP010399.1), p6411-9.012 kb (CP010370.2), pNDM-BJ01 (JQ001791.1), pNDM-lz4b (KJ547696.1), and pNDM-JVAP01 (KM923969.1). All of them harbor the genetic structure IS*Aba14-aph(3’)-VI*-IS*Aba125-bla*_NDM-1_-*ble-MBL* (Fig. 5). These plasmids were isolated from patient or environmental strains belonging to the *Acinetobacter* genus, including *A. baumannii*, *A. lwoffii*, *A. nosocomialis*, and *A. lactucae*. These findings suggest that the *bla*_NDM-1_ gene may be widely disseminated among *Acinetobacter* species via these plasmids. *bla*_OXA-58_ on pXH1041 is found with a single copy of IS*Aba3*, whereas *bla*_OXA-24_ on pXH1032 is not associated with any insertion sequence. pXH1032 is a 7,852-bp plasmid carrying the *bla*_OXA-24_ resistance gene isolated from Guangdong (China), and BLAST analysis showed that it was identical to plasmid pA1429a (CP046901.1) isolated from Hangzhou (China). This sequence was also similar to the backbone of p3UC20804 (CP076810.1), which was isolated first in Chile and did not carry any resistance gene, and pIP1858 (KP890934.1), which was isolated in France and carried *aac (6')-Ih* (Fig. 6). The plasmid of strain XH1041 was highly similar to that of pBJAB0715 (CP003848.1) carrying *bla*_OXA-58,_*aph (3')-VIa* and *aac (3)-IId*. The genetic structure of the *bla*_OXA-58_ gene of pXH1041 is ISOur1-ΔIS*Aba3*-like element-*bla*_OXA-58_- IS*Aba3*, which is identical to that of plasmid pWH8144 (Q241792.1) (Fig. 7) (37). The two strains not listed in Table 1, XH1022 and XH1034, do not carry any carbapenemase-encoding genes, which is also consistent with their susceptibility phenotypes.

**TABLE 1:** Isolates carrying acquired carbapenem resistance genes, their localization, copy number, MIC of meropenem and imipenem, and their gene environment and structure

Carbapenemase	Isolate	Gene localization	Copy number	MEM MICs	IPM MICs	Associated mobile element	Tandem structure
*bla*_OXA-23_	XH1018	Chromosome	1	32	32	Tn*2009*	/^*b*^
	XH1019	Plasmid	1	64	32	Tn*2009*	
	XH1026	Plasmid	1	64	32	Tn*2009*	
	XH1033	Chromosome	1	32	32	Tn*2009*	
	XH1035	Plasmid	1	64	64	Tn*2009*	
	XH1036	Plasmid	1	64	128	Tn*2009*	
	XH1037	Chromosome	1	32	32	Tn*2008*	
	XH1039	Chromosome	1	32	32	Tn*2009*	
	XH1040	Plasmid	1	64	128	Tn*2009*	
	XH1042	Plasmid	1	64	64	Tn*2009*	
	XH1043	Chromosome	1	32	32	Tn*2009*	
	XH1044	Plasmid	1	64	32	Tn*2009*	
	XH1045	Plasmid	1	64	32	Tn*2009*	
	XH1048	Plasmid	1	64	>128	Tn*2009*	
	XH1050	Chromosome	1	32	32	Tn*2009*	
	XH1054	Plasmid	1	>128	128	Tn*2009*	
	XH1055	Plasmid	1	64	64	Tn*2009*	
	XH1056	Chromosome	1	32	32	Tn*2009*	
	XH1015	Chromosome	2	64	64	Tn*2009*	Yes
	XH1017	Chromosome	2	64	64	Tn*2009*	No
	XH1023	Chromosome	2	64	64	Tn*2006*	No
	XH1024	Chromosome	2	64	64	Tn*2006*	Yes
	XH1041	Chromosome	2	64	32	Tn*2009*	Yes
	XH1047	Chromosome	2	64	32	Tn*2009*	Yes
	XH1051	Chromosome	2	32	32	Tn*2009*	Yes
	XH1052	Chromosome	2	64	32	Tn*2009*	No
	XH1016	Chromosome	4	>128	128	Tn*2009*	Yes
*bla*_OXA-24_	XH1032	Plasmid	1	64	128	ND^*a*^	/
*bla*_NDM-1_	XH1038	Plasmid	1	>128	>128	IS*Aba125*	/
*bla*_OXA-58_	XH1041	Plasmid	1	64	32	IS*Aba3*	/

^*a*^ "ND”-indicates that no relevant mobile element was detected.

^*b*^ "/"- indicates the presence of only a single-copy resistance gene, which is not statistically significant.

### Comparison of resistance rates of isolates in ST2 vs non-ST2 and MTE vs non-MTE groups

The 45 strains of this panel were identified by MLST, and a total of 28 strains (62.2%, 28/45) belonged to ST2 (Pasteur scheme) isolates, which showed significantly higher resistance to cefepime, ceftazidime, imipenem, meropenem, ciprofloxacin, amikacin, and gentamicin than the non-ST2 group, whereas there was no difference in the resistance to colistin, tigecycline, and eravacycline among the isolates (Table 2). The morphology of the colonies on sheep blood plates also demonstrated the diversity of the panel. The panel strains were classified into three different types of groups according to previous descriptions (11), of which seven were MTA with an "oil on water" phenotype, four were MTC with a "raised center and irregular base" phenotype, and 34 were MTE with a "flat center and wavy edges" phenotype. In order to allow for intergroup comparisons, we chose to compare the MTE group with the non-MTE group. The MTE isolates differed only in their resistance to eravacycline, with none of the strains being resistant to eravacycline (*P* = 0.002), and there was no difference in resistance to any of the other antibiotics tested between the two groups (Table 3).

**TABLE 2:** Comparison of ST2 and non-ST2 resistance rates in *A. baumannii*

	ST2 (*n* = 28)		Non-ST2 (*n* = 17)		
	MIC range(mg/L)	Resistance rate (100%)	MIC range(mg/L)	Resistance rate (100%)	*P* value
FEP	32 to ＞256	100%	4 to ＞256	82.40%	0.048
CAZ	2 to ＞256	96.40%	2 to ＞256	58.80%	0.003
IPM	32 to 128	100%	0.125 to ＞128	82.40%	0.048
MEM	32 to ＞128	100%	0.25 to ＞128	82.40%	0.048
CIP	4 to ＞32	100%	＜0.0625 to ＞32	64.70%	0.002
AK	＜0.25 to ＞256	92.90%	＜0.25 to ＞256	52.90%	0.003
GEN	1 to ＞512	96.40%	＜0.25 to ＞512	52.90%	0.001
COL	＜0.03125 to 1	0%	0.0625 to 2	0%	1
TGC	0.5 to 8	25.00%	0.25 to 4	5.90%	0.132
ERA	＜0.03125 to 2	10.70%	＜0.03125 to 1	5.90%	1

**TABLE 3:** Comparison of MTE and non-MTE resistance rates in *A. baumannii*

	MTE (*n* = 34)		Non-MTE (*n* = 11)		
	MIC range (mg/L)	Resistance rate (100%)	MIC range (mg/L)	Resistance rate (100%)	*P* value
FEP	4 to >256	94.12%	4 to >256	90.91%	1
CAZ	2 to >256	79.41%	2 to >256	90.91%	0.657
IPM	0.125 to >128	94.12%	0.25 to 64	90.91%	1
MEM	0.25 to >128	94.12%	0.5 to 64	90.91%	1
CIP	<0.0625 to >32	85.29%	<0.0625 to >32	90.91%	1
AK	<0.25 to >256	79.41%	<0.25 to >256	72.73%	0.687
GEN	<0.25 to >512	82.35%	<0.25 to >512	72.73%	0.666
COL	<0.03125 to 2	0.00%	0.0625 to 2	0%	1
TGC	0.125 to 8	17.65%	0.5 to 4	18.18%	1
ERA	<0.03125 to 0.5	0.00%	<0.03125 to 2	36.36%	0.002

### Heterogeneity of biofilm formation and growth rates

**Fig 8:**
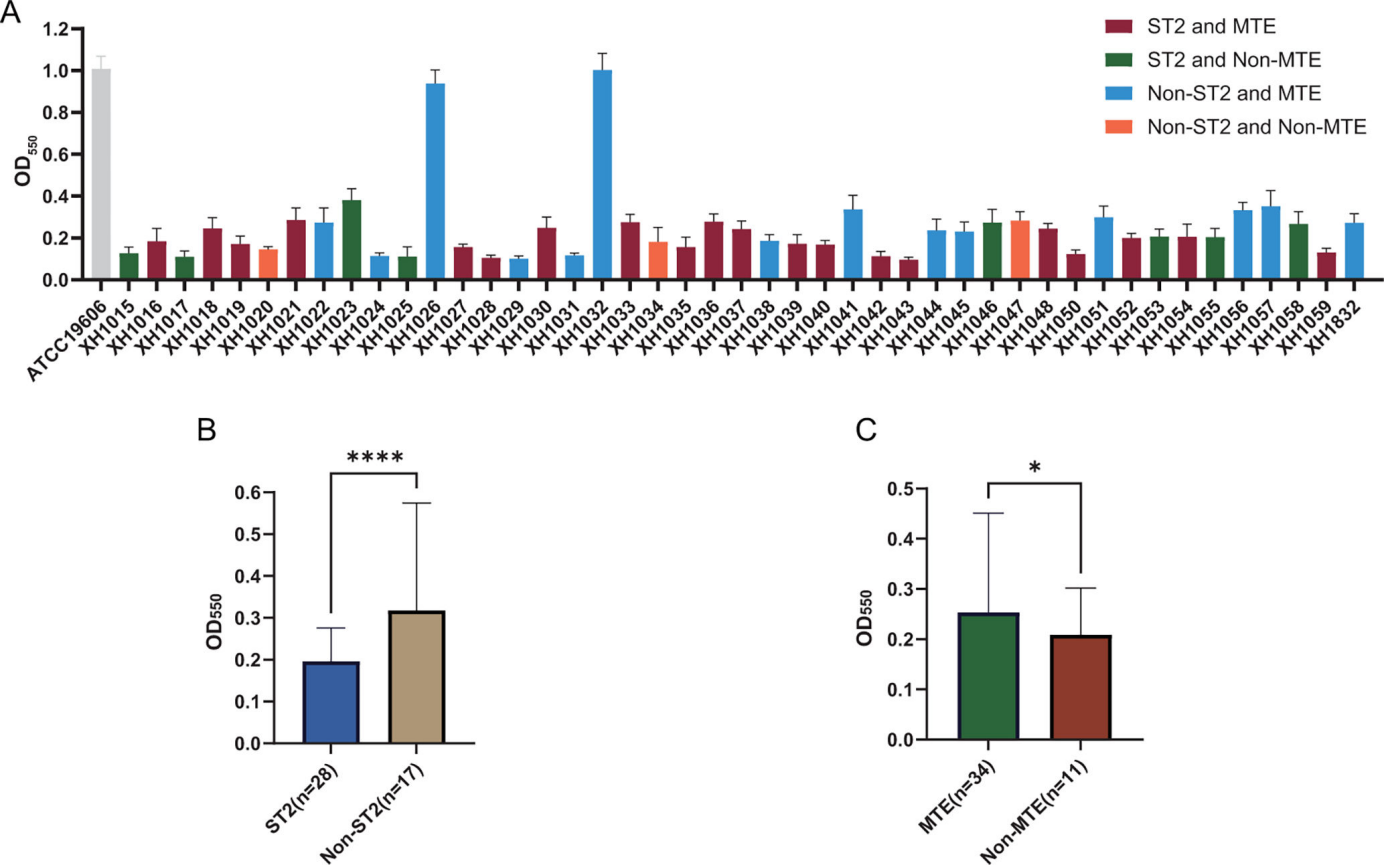
Biofilm formation capacity. (**A**) Biofilm formation capacity of panel strains; (**B**) comparison of biofilm formation in ST2 versus non-ST2; (**C**) comparison of biofilm formation in MTE versus non-MTE. Data were analyzed by independent samples *t*-test and expressed as mean ± standard deviation, with asterisks indicating significant differences between groups (*P < 0.05, **P < 0.01, **P < 0.001, and ****P < 0.0001).

**Fig 9:**
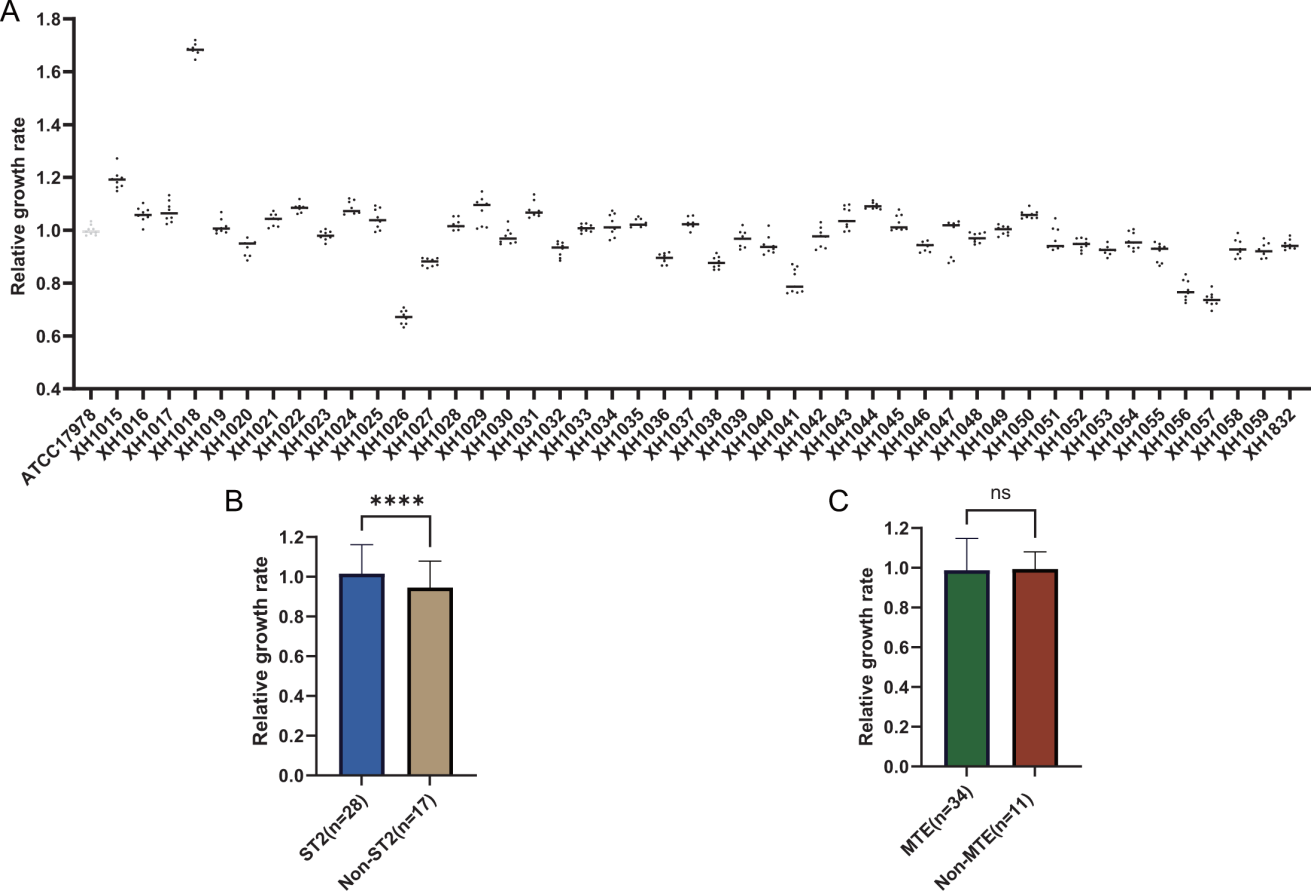
Growth rates. (**A**) Growth rates of panel strains; (**B**) comparison of ST2 and non-ST2 growth rates; (**C**) comparison of MTE and non-MTE growth rates. Data were analyzed by independent samples *t*-test and expressed as mean ± standard deviation, with asterisks indicating significant differences between groups (*P < 0.05, **P < 0.01, **P < 0.001, and ****P < 0.0001).

The assay data showed that all the strains had the ability to form biofilms, but each strain differed in its biofilm-forming ability, and heterogeneity was observed. Of particular interest is that XH1026 and XH1032 produced biofilms at a level comparable to that of the reference strain ATCC19606. ST2 isolates produced low biofilms, whereas MTE-type isolates produced strong biofilms (Fig. 8). In addition, the growth rates of all strains were highly variable, with rates in MHB (OD_600_) ranging from 0.67 to 1.68. XH1018 showed minimal cost of fitness, i.e., a very rapid growth rate in the absence of antibiotic stress, whereas the relative growth rates of XH1026, XH1056, and XH1057 were very low. There was a significant difference in growth rates between isolates of ST2 and non-ST2, and there was no significant difference between groups with different colonial morphologies (Fig. 9).

### Virulence phenotypes and relationship to specific KL types

**Fig 10:**
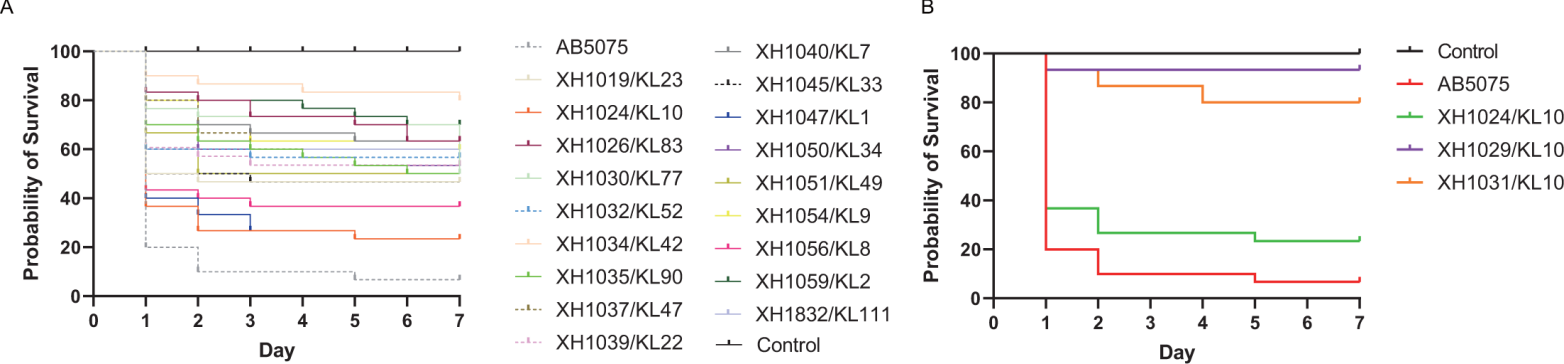
(**A**) *In vivo* virulence of *A. baumannii* of different KL types in he *G. mellonella* model; (**B**) Results of *in vivo* virulence of KL10 isolates of the *G. mellonella* model. X-axis, days after inoculation; y-axis, *G. mellonella* survival rates. PBS without bacteria was used as a negative control, and AB5075 was used as a positive control.

Next, we performed *in vivo* virulence assays in *Galleria mellonella* on all different KL isolates. All tested *A. baumannii* strains exhibited less virulence than the positive virulence control AB5075, and within this, XH1024 and XH1047 were more virulent (Fig. 10A). To test the hypothesis if the capsule type correlates with virulence, we tested the virulence of three KL10 strains, XH1024, XH1029, and XH1031. The results showed that the virulence of XH1029 and XH1031 was very low when compared to that of XH1024, illustrating the heterogeneity among strains, even in strains of the same KL type (Fig. 10B).

## DISCUSSION

*A. baumannii* is a common clinical pathogen that is easily transmitted in hospitals, causing a variety of serious infections due to its persistence in the environment and acquired multidrug resistance (38). In recent years, the rate of *A. baumannii*-associated infections, antibiotic resistance, and morbidity has been increasing, placing a serious burden on the global healthcare system (39). In order to provide protection against *A. baumannii* infections, researchers are actively exploring vaccines, antimicrobial peptides, phage therapeutics, or novel antimicrobial drugs (40–44). However, at this stage, the test strains used to evaluate these therapeutic candidates vary from study to study, coming from different sources and in varying numbers (often very large and with very little heterogeneity), which undoubtedly complicates the complexity of the studies, and more importantly, makes it difficult to establish the general effectiveness of the new treatment/diagnostic. In addition, there are a considerable number of studies on *A. baumannii* pathogenesis and drug therapy that use reference strains only. However, it has been shown that these long-established and widely used “reference,” namely, ATCC19606 (ST52/KL3), ATCC17978 (ST437/KL3), and AB5075 (ST1/KL25), belong to rare sequence types. Their colony morphology and capsule formation are also limited compared to many other *A. baumannii* isolates (11). Thus, they cannot represent current clinical strains in terms of their phenotypic and genotypic diversity. Therefore, the research community would benefit from a standardized, heterogeneous panel of strains.

To the best of our knowledge, there were three *A. baumannii* panel repositories. The first one is a panel of 41 strains published jointly by the Centers for Disease Control and Prevention and Food and Drug Administration (CDC-FDA) (45); the second one is a panel of *Acinetobacter* spp. containing eight *A*. *baumannii* strains collected from a hospital in Seoul in South Korea (46); and the third one is a panel of 100 *A*. *baumannii* strains developed by the Multidrug-Resistant Organism Repository and Surveillance Network (MRSN) of the Walter Reed Army Research Institute in the United States (47). However, all of them have some shortcomings to a greater or lesser extent: all of them lack the phenotypic results of the strains. Although all the strains included in this panel were collected in China, it has representatives of the globally distributed clones, ST208_OXF_, ST191_OXF_, ST368_OXF,_ and ST369_OXF_ (48). These strains possess prevalent acquired resistance genes such as *bla*_OXA-23_, *bla*_OXA-24_, and *bla*_OXA-58_, as well as the intrinsic OXA-51-like carbapenemases *bla*_OXA-66_ and *bla*_OXA-69_ (49). Furthermore, there are emerging sequence types, including ST452_OXF_, ST684_OXF_, and ST373_OXF_ (50, 51). Previous studies established that KL2, KL10, KL22, and KL52 were the major types among the carbapenemase-resistant *A. baumannii* (CRAB), while also leading to more severe infections (especially pneumonia) and higher mortality (52). Several studies also reported KL49 to be a phenotype with higher virulence and clinical mortality (53). All of these capsular types were included in this study, which also provides a good resource for conducting capsular-related studies. Furthermore, a strain which harbors a novel resistance island AbGRI5 is the isolate XH1056, found in our panel (54). One major advantage of our panel is that we characterized the multiple phenotypes of the strains, allowing informed selection of strains based on specific biological questions.

ST2 *A. baumannii* is prevalent worldwide with multiple resistance genes and resistance plasmids, leading to wide dissemination of resistance; thus, ST2 is strongly correlated with resistance to a variety of antibiotics, such as cephalosporins, carbapenems, quinolones, and aminoglycosides (55). In addition to antimicrobial resistance, ST2 isolates commonly exhibit strong biofilm-forming capacity, which can increase their pathogenicity (56). In our study, ST2 was the most common sequence type, and ST2 isolates showed significantly higher rates of resistance to cefepime, ceftazidime, imipenem, meropenem, ciprofloxacin, amikacin, and gentamicin, while also not showing a strong loss of fitness, i.e., a lower cost of adaptation. Unsurprisingly, *A. baumannii* ST2 can be clinically predominant. Surprisingly, the ST2 strains in our panel produced lower biofilms than those in the non-ST2 group (57). However, by comparing the laboratory characteristics of IC2 and non-IC2 *A. baumannii* isolates, the biofilm-forming ability of IC2 was found to be significantly lower than that of non-IC2 isolates (58), which is similar to our findings. Szczypta *et al*. collected all strains from two clonal outbreaks in the same hospital and showed that all ST2 strains tested had lower biofilm formation capacity than ATCC19606, which belongs to ST52 (59). Interestingly, previous studies observed that many multidrug resistant clinical isolates did not form biofilms. In contrast, a large number of strong biofilm producers were drug-sensitive *A. baumannii* strains; the biofilm can protect the bacteria by forming a barrier, preventing many antibiotics to reach the cells embedded within the biofilm (60, 61). Research published to date has yielded confusing perspectives on the relationship between biofilms and bacterial resistance to antibiotics, with some studies indicating a positive correlation, others a negative correlation, or no relationship at all (62–64). Studies have reported that strains from ICU patients and non-burn patients produce more biofilms; furthermore, bacteria from clinical environments produce more biofilms than those from environmental sources (61, 65, 66). In fact, the formation of biofilms by strains is a rather complex process that not only accounts for individual differences among different strains but also takes into consideration other factors such as incubation time, nutritional media, and staining time. When examining the methodologies regarding biofilms, it is apparent that there are variations in experimental conditions, and there is no clear standard for the absorbance value that determines whether the strains studied form biofilms (61). Consequently, relying solely on biofilm formation to predict antimicrobial resistance is one-sided and lacks credibility. These findings suggest that when exploring the relationship between biofilm formation and antimicrobial resistance, it is important to consider the source of the strain and the influence of external environments, as these may affect the final conclusions. Previous studies have observed the presence of genes such as *bla*_PER-1_, *bla*_TEM_, *tetB, bla*_oxa_, and class 1 integrons in biofilm-producing strains (67, 68), which may provide an alternative explanation for the relationship between biofilms and antimicrobial resistance, and further studies are needed to elucidate the mechanisms involved in this process. Given the spread of *A. baumannii* ST2 strains, especially in clinical settings, it is important to monitor the prevalence also to establish effective measures to limit the distribution of such strains.

It has been shown that hypervirulent *K. pneumoniae* (hvKp) tends to exhibit a hypermucoviscous phenotype. In addition, studies have reported the emergence and spread of carbapenem-resistant *K. pneumoniae* (CRKP) with a novel "red, dry, and rough" morphology (rdar) (69). In the case of *A. baumannii*, multi-antibiotic resistance has also been reported for the mucoid phenotype (70). These studies suggest a relationship between colony morphology and bacterial antibiotic resistance and virulence. Differences in colony morphology may also potentially reflect differences in antibiotic resistance and virulence of strains. We divided the strains in this panel into three groups, as described in the literature (11), and further investigated the relationships between the MTE "flat center and wavy edge" phenotype with antibiotic resistance, biofilm, and growth rates. We found that strains with the MTE phenotype were more resistant to eravacycline and produced less biofilms, while there was no significant difference in their growth rates. However, due to the limited number of strains, the relationship between MTE phenotypes and bacterial antibiotic resistance, as well as biofilm production, needs to be investigated more thoroughly by increasing the number of samples.

The virulence phenotypes of the strains tested in this study also exhibit diversity, ranging from low to high virulence. In the future, these strains can be employed for virulence research, expanding beyond the use of just strain AB5075. Our data also indicate that there is no clear correlation between the KL type of A. *baumannii* and the strain’s virulence. The virulence of *A. baumannii* is not only determined by a single factor but by many (71), which makes it impossible to link pathogenicity to a specific KL type.

One remaining issue with our panel is that our study does not include pan-resistant strains and also no colistin-resistant strains. Being limited in the number of isolates, our panel can of course not cover all of the clonal lineages, which is the case in any panel that exists to date. Thus, with the large number of strains globally, the constant emergence of novel clones, and the constant evolution of strains, it is impossible to completely cover all clones. Nonetheless, our research can be beneficial for the development of vaccines or for the isolation of novel phages for phage therapy (72, 73). Nonetheless, this panel covers the major and most of the currently clinically problematic *A. baumannii* clones.

In reality, one significant factor contributing to the circulation of various variants of reference strains among laboratories is improper handling of these strains. The ATCC clearly stipulates that strain passages should not exceed five generations (https://www.atcc.org/resources/technical-documents/reference-strains-how-many-passages-are-too-many). However, most researchers are not fully cognizant of it and often distribute strains to other laboratories without proper consideration. Researchers who receive these strains through such exchanges continue to distribute them indiscriminately to other colleagues. Over time, these strains undergo numerous passages, leading to genomic rearrangements. Consequently, it is plausible that different laboratories end up with multiple variants of the reference strains. To address this issue, we recommend that all individuals using strains from this panel take steps to prevent multiple passages, thereby preventing the appearance of more variants. It is crucial to adhere to proper strain management practices to maintain the integrity and reliability of the reference strains for accurate scientific research and testing. We believe that our panel of 45 different strains of *A. baumannii* from a collection of 2,197 *A*. *baumannii* will facilitate multiple medical, scientific, and translational studies to combat infections caused by this critical pathogen.

## Data Availability

Genotypic and phenotypic data for the diverse panel of 45 *A*. *baumannii* strains are available at http://phdb.baumanii.dmicrobe.cn/. These strains will be submitted to the Guangdong Microbial Culture Collection Center (GDMCC, https://gdmcc.net/#/index) for public storage, and interested applicants can apply to the GDMCC for free access to all strains. Raw sequence data for all isolates are available through the National Center for Biotechnology Information under the BioProject accession number PRJNA1016089.

## SUPPLEMENTAL MATERIAL

Fig. S1 spectrum.00086-24-s0001.pdf Colony morphology of panel strains grown on Columbia AGAR containing 5% sheep blood.

ASM does not own the copyrights to Supplemental Material that may be linked to, or accessed through, an article. The authors have granted ASM a non-exclusive, world-wide license to publish the Supplemental Material files. Please contact the corresponding author directly for reuse.
