# Supplementary material for: A panel of genotypically and phenotypically diverse clinical Acinetobacter baumannii strains for novel antibiotic development
Source: Microbiol Spectr. 2024 Jun 25;12(8):e00086-24. doi: 10.1128/spectrum.00086-24 (PMC11302250; doi:10.1128/spectrum.00086-24)

Supplementary Material 1. Colony morphology of panel strains grown on Columbia AGAR containing 5% sheep blood.

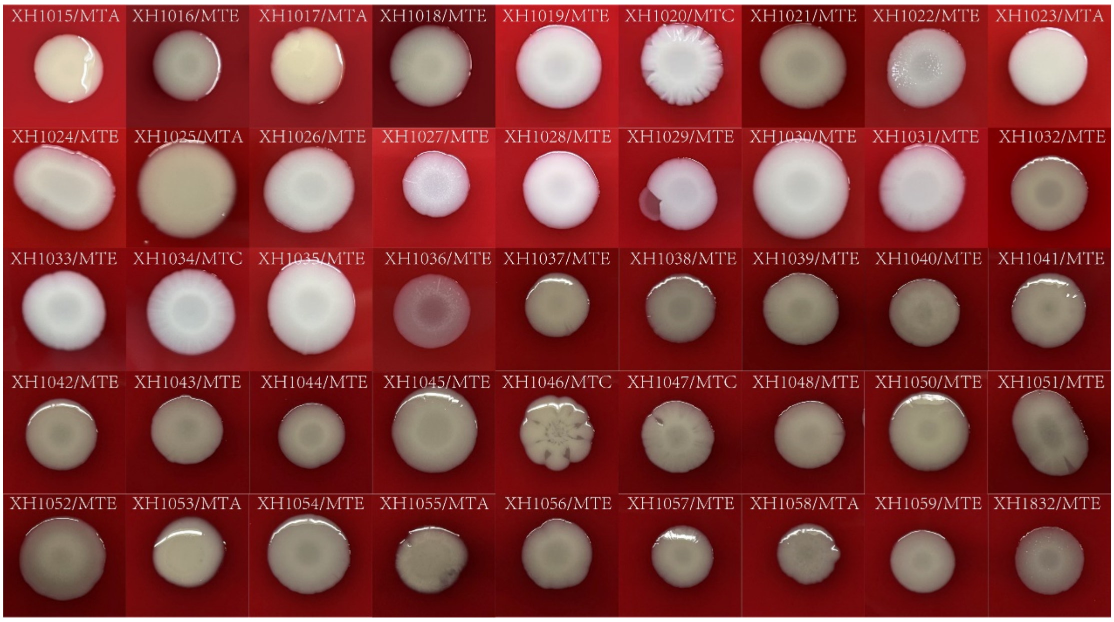

Supplement: Fig. S1 — Colony morphology of panel strains grown on Columbia AGAR containing 5% sheep blood. [file spectrum.00086-24-s0001.pdf]
